# MiR-424-5p regulates cell cycle and inhibits proliferation of hepatocellular carcinoma cells by targeting E2F7

**DOI:** 10.1371/journal.pone.0242179

**Published:** 2020-11-17

**Authors:** Yichao Zhao, Chaoqian Zhu, Qing Chang, Peng Peng, Jie Yang, Chunmei Liu, Yang Liu, Xiaonan Chen, Yuanguang Liu, Ran Cheng, Yijie Wu, Xiaotang Wu, Liang Hu, Jun Yin

**Affiliations:** 1 Department of Hepatobiliary Surgery, Tangshan Gongren Hospital, Tangshan, China; 2 Department of Head and Neck Surgery, Tangshan Gongren Hospital, Tangshan, China; 3 Department of Geriatrics, Tangshan Gongren Hospital, Tangshan, China; 4 Shanghai Engineering Research Center of Pharmaceutical Translation, Shanghai, China; Universitat des Saarlandes, GERMANY

## Abstract

**Objective:**

This study aims to explore the mechanism of the miR-424-5p/E2F7 axis in hepatocellular carcinoma (HCC) and provide new ideas for targeted therapy of HCC.

**Methods:**

Bioinformatics analysis was used to identify the target differentially expressed miRNA in HCC and predict its target gene. qRT-PCR was employed to verify the expression of miR-424-5p and E2F7 mRNA in HCC cells. Western blot was performed to detect the effect of miR-424-5p ectopic expression on the protein expression of E2F7. CCK-8 was used to detect proliferative activity of HCC cells and flow cytometry was carried out for analyzing cell cycle distribution. Dual luciferase reporter assay was conducted to verify the direct targeting relationship between miR-424-5p and E2F7.

**Results:**

We observed that miR-424-5p was down-regulated in HCC cells. CCK-8 showed that overexpression of miR-424-5p inhibited cell proliferation, and flow cytometry showed that miR-424-5p could block cells in G0/G1 phase. E2F7 was up-regulated in HCC cells, and E2F7 overexpression could facilitate the proliferative ability of HCC cells and promote the cell cycle progressing from G0/G1 to S phase. Furthermore, dual-luciferase reporter assay indicated that miR-424-5p could directly down-regulate E2F7 expression. Analysis on cell function demonstrated that miR-424-5p inhibited the proliferation of HCC cells and blocked cell cycle at G0/G1 phase by targeting E2F7.

**Conclusion:**

Our results proved that E2F7 was a direct target of miR-424-5p, and miR-424-5p could regulate cell cycle and further inhibit the proliferation of HCC cells by targeting E2F7.

## Introduction

The mortality rate of hepatocellular carcinoma (HCC) ranks the third among malignant tumors in the world, with about 1 million new cases diagnosed each year, and the incidence rate of HCC continues to rise [1,2]. Due to the late diagnosis, drug resistance, tumor recurrence and metastasis, etc., the 5-year overall survival (OS) rate of HCC is low of approximately 7% [3,4]. Up to present, surgical resection, liver transplantation and percutaneous ablation are still the main treatment strategies for HCC, yet they are only suitable for some early stage patients. Besides, owing to imperceptible symptoms of HCC at early stage, most patients are diagnosed at advanced stage and are not eligible for the abovementioned local treatments. Therefore, elucidating the molecular mechanism of HCC will contribute to the development of new therapies for HCC to improve the OS rate.

MicroRNAs (miRNAs) were first discovered in 1993, and some specific miRNAs have been found to be involved in crucial biological processes such as growth, cell proliferation, apoptosis and carcinogenesis after years of research [5–7]. Moreover, miRNAs in circulatory system can be stably detected in serum and plasma, and are expected to be noninvasive biomarkers for early diagnosis and prognosis of cancer [8,9]. Many studies have reported the abnormal expression and biological function of miRNAs in liver cancer. For example, miR-486 is obviously down-regulated in liver cancer, and its ectopic expression can hinder the occurrence of tumor [10]. MiR-498 inhibits growth and metastasis of liver cancer by targeting and down-regulating the expression of ZEB2 [11]. MiR-222 inhibitor may have an anti-tumor effect on liver cancer cells by binding to 3’-UTR of BBC3 [12]. MiR-424-5p is located on human chromosome Xq26.3, and recently has been classified into a large cluster together with miR-15/miR-16 [13]. However, the expression of miR-424-5p in different tumor types suggests unequal roles. Recent studies have shown that miR-424-5p is down-regulated in cancers including intrahepatic cholangiocarcinoma, esophageal squamous cell carcinoma and epithelial ovarian cancer [14–16], and inhibits proliferation and metastasis of cancer cells. While, Yujun Li *et al*. [17] showed that miR-424-5p stimulates the proliferation, migration and invasion of laryngeal squamous cell carcinoma. However, the underlying molecular mechanism and the specific biological function of miR-424-5p in HCC have not been fully elucidated. Therefore, studying the mechanism of miR-424-5p in HCC is beneficial for the development of new strategies for the prognosis and treatment of HCC.

In order to fully elucidate the mechanism of miR-424-5p in HCC, bioinformatics analysis was used to predict the novel downstream target E2F7 of miR-424-5p, and the expression patterns of miR-424-5p and E2F7 in HCC and their effects on proliferation were investigated. Finally, rescue experiments were performed to explore the role of the miR-424-5p/E2F7 regulatory axis in HCC.

## 1 Materials and methods

### 1.1 Bioinformatics analysis

Expression profiles of miRNA (normal: 50; tumor: 375) and mRNA (normal: 50; tumor: 374) in TCGA-LIHC were mined from TCGA database (http://www.tcga.org/). “edgeR” package was employed to perform differential analysis on miRNAs and mRNAs with |logFC|>1.5 and padj<0.05 as the threshold. TargetScan (http://www.targetscan.org/), miRDB (http://mirdb.org/) and mirTarBase (http://mirtarbase.cuhk.edu.cn/php/index.php) databases were used to conduct target gene prediction for the identified upstream regulator miRNA. The predicted target genes were intersected with the differentially expressed mRNAs (DEmRNAs). A total of 370 tumor samples with both mRNA and miRNA sequencing data were included for Pearson correlation analysis, and the paired *t*-test was used for statistical significance assessment. According to the median value of the target gene expression level in all tumor samples, samples were divided into high/low expression groups. Kaplan Meier method was used to analyze the survival between the two groups, and log rank was used for statistical analysis.

### 1.2 Cell culture and transfection [18]

Human HCC cell lines Hep 3B (HB-8064), HEP G2 (HB-8065), SNU-182 (CRL-2235), SNU-387 (CRL-2237), human normal liver cell line THLE-3 (CRL-11233) and the HEK293t cell line used for dual-luciferase reporter assay were cultured in Dulbecco’s Modified Eagle’s Medium (DMEM; Gibco, NY) containing 10% fetal bovine serum (FBS), 100 U/mL penicillin and 100 μg/mL streptomycin. Cells were placed in an incubator at 37°C with 5% CO_2._ Above cell lines and medium were purchased from American Type Culture Collection (ATCC; Manassas, VA, USA).

For cell transfection, HCC cells were seeded onto 6-well plates with a density of 2×10^5^ cells/well. When the cell confluence reached to 80%, miR-424-5p mimic (miR-mimic) or corresponding control (NC-mimic) and E2F7 overexpression plasmids (oe-E2F7) or empty vector control (oe-NC) were respectively transiently transfected into target cells using lipofectamin2000 (Invitrogen, Carlsbad, USA) according to instructions. Medium was replaced after 6 h and cells were collected after 48 h of transfection. MiR-mimic and NC-mimic were purchased from GenePharma (Shanghai, China), while oe-E2F7 and oe-NC were purchased from RiboBio company (Guangzhou, China).

### 1.3 qRT-PCR [19]

Total RNA was extracted from cells using TRIzol reagent kit (Invitrogen) according to the instructions and purified by phenol/chloroform. cDNA was synthesized via reverse transcription. qRT-PCR was performed on the ABI 7500 HT Fast Real-Time PCR System (Applied Biosystems, CA, USA) using the SYBR Green PCR Master Mix (Thermo Fisher Scientific, MA, USA). The relative expression of miRNA and mRNA was normalized to U6 and GAPDH, respectively. The quantitative value of the relative expression of miRNA and mRNA was analyzed by 2^-ΔΔCT^ method. All PCR primer sequences in the experiment were as follow: miR-424-5p forward: 5'-GCCAGCAGCAATTCATGT-3', miR-424-5p reverse: 5'-TATGGTTTTGACGACTGTGTGAT-3'; U6 forward: 5'-ATTGGAACGATACAGAGAAGATT-3', U6 reverse: 5'-GGAACGCTTCACGAATTTG-3'; E2F7 forward: 5'-TGTGAGCTATCTGGAAGAACC-3', E2F7 reverse: 5'-TTCAGTCCGACTGGTCACTCA-3'; GAPDH forward: 5'-ACCCAGAAGACTGTGGATGG-3', GAPDH reverse: 5'-TTCTAGACGGCAGGTCAGGT-3'.

### 1.4 CCK-8

The cell proliferative ability was measured using cell counting kit 8 (CCK-8) (Beyotime Biotechnology, Shanghai, China). Cells in different treatment groups were planted into 96-well plates at a density of 3×10^3^ cells/well. In accordance with the specifications, 10 μL of CCK-8 solution was added into each well. Optical density (OD) values at 0, 24, 48, 72, and 96 h at 450 nm were recorded using a spectrophotometer. Each experiment was conducted in triplicate.

### 1.5 Flow cytometry [20]

Cells were harvested and digested with trypsin, and about 1×10^6^ cells were used for cell cycle analysis. Cells were washed with PBS and fixed in 70% ice-cold ethanol overnight at 4°C, followed by washing with PBS and culture in 1 mL of staining solution (20 mg/mL propidium iodide, 10 U/mL RNaseA) for 30 min at room temperature. The DNA content was analyzed using a flow cytometry on the FACSCalibur system (Becton Dickinson), and the cell cycle distribution was analyzed.

### 1.6 Western blot

After transfection for 48 h, proteins were extracted from cultured cells using RIPA buffer (Thermo Fisher Scientific) containing protease inhibitor. Then, the protein concentration was assayed by BCA protein assay kit (Thermo Fisher Scientific). The high-temperature denatured protein samples were separated by sodium dodecyl sulfate-polyacrylamide gel electrophoresis (SDS-PAGE) at 100 V and transferred onto the polyvinylidene fluoride (PVDF) membranes (Amersham, USA). The membranes were incubated with primary antibodies overnight at 4°C after being blocked for 1 h. Then, the membranes were incubated with horseradish peroxidase labeled secondary antibody goat anti-rabbit IgG H&L (ab97051, 1:2000, Abcam, Cambridge, UK) at room temperature for 1 h. Afterwards, the membranes were washed with TBST buffer for three times. The primary antibodies included rabbit polyclonal anti-E2F7 (ab56022, 1:1000, Abcam) and rabbit polyclonal anti-GAPDH (ab9484, 1:1000, Abcam). All proteins were visualized using an optical luminometer (GE, USA). The relative expression of proteins was analyzed by using the Image Pro Plus 6.0 (Media Cybernetics, USA). All experiments were conducted in triplicate.

### 1.7 Dual luciferase reporter gene assay [18]

The human wild-type (Wt) and mutant-type (Mut) E2F7 3’-UTR sequences were synthesized and cloned into the pMIR-GLO luciferase vectors to construct pMIR-E2F7-3’-UTR-Wt (E2F7-Wt) and pMIR-E2F7-3’-UTR-Mut (E2F7-Mut). E2F7-Wt or E2F7-Mut along with miR-424-5p mimic or NC mimic was transfected into HEK293t cells, and the confluence rate could reach to 60%–80%. The luciferase activities were measured using the dual-luciferase reporter assay system (Promega, Madison, WI, USA) 24 h after transfection. Transfection efficiency was normalized based on the firefly luciferase activity and the Renilla luciferase activity.

### 1.8 Statistical analysis

All experiments were performed in triplicate, and data were presented as mean ± standard deviation. Statistical analyses were conducted using SPSS21.0 software (SPSS, Inc, Chicago, USA). Student’s *t*-test was used to analyze the differences between two independent groups, while one-way ANOVA method was used to evaluate the differences among multiple groups. *P*<0.05 was considered statistically significant difference.

## 2 Results

### 2.1 MiR-424-5p is significantly down-regulated in HCC tissue and cells, while overexpression of miR-424-5p inhibits proliferation of HCC cells

**Fig 1:**
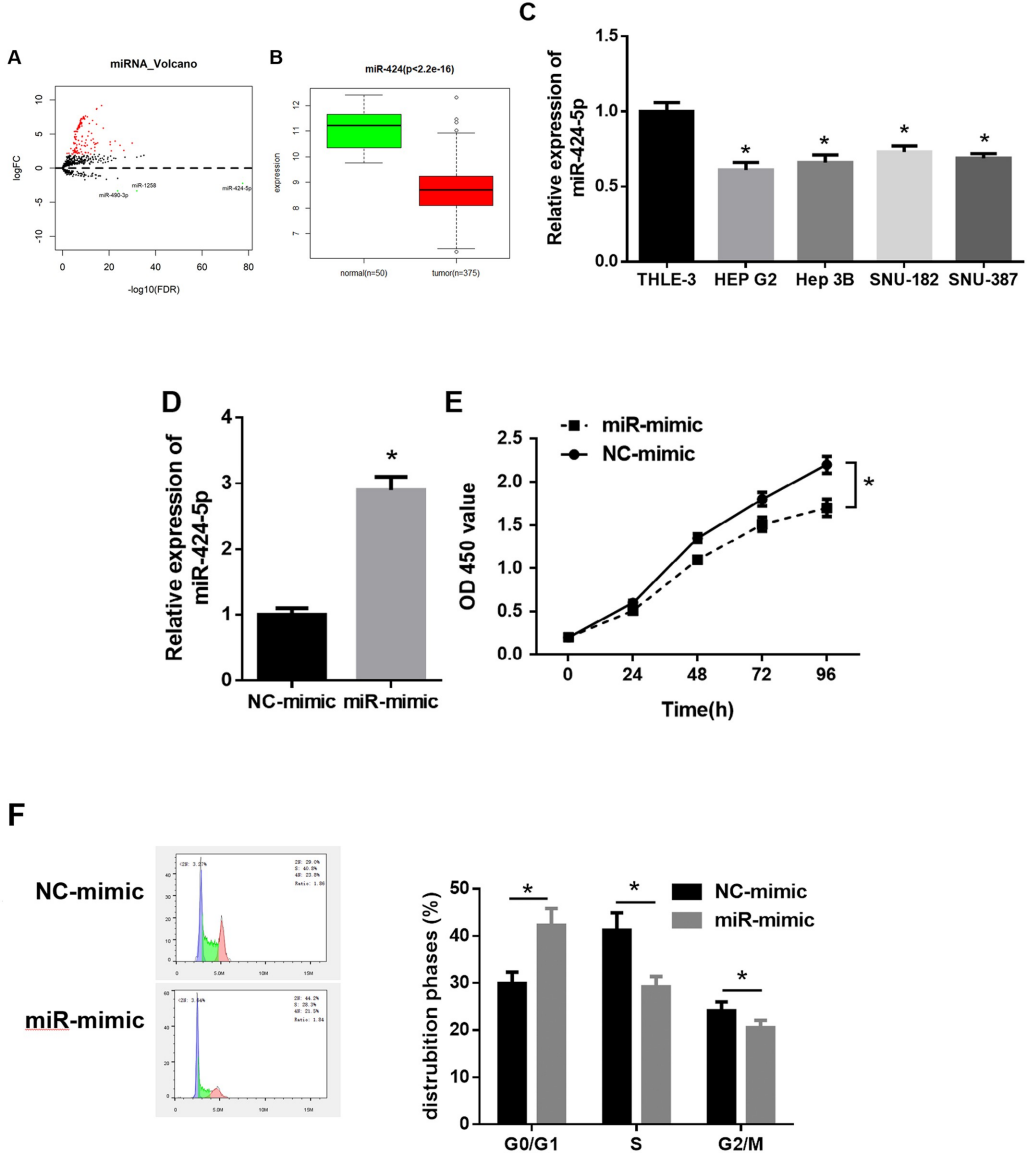
MiR-424-5p expression level and role in HCC cells. A: Volcano plot of DEmiRNAs in normal group and tumor group of HCC from TCGA database; B: Box plot of miR-424 expression in normal group and tumor group; C: qRT-PCR was used to detect the expression of miR-424-5p in normal THLE-3 cell line and HCC cell lines (HEP G2, Hep 3B, SNU-182 and SNU-387); D: qRT-PCR was performed to detect the transfection efficiency of miR-424-5p mimic and NC-mimic; E: CCK-8 assay was used to determine the effect of miR-424-5p overexpression on the proliferative ability of HEP G2 cells; F: Flow cytometry was used to analyze cell cycle; * *p*<0.05.

Differential analysis was performed on miRNA expression data of HCC obtained from TCGA, and 127 differentially expressed miRNAs (DEmiRNAs) were screened, of which miR-424 was significantly down-regulated in tumor tissue of HCC (Fig 1A and 1B). MiR-424 has been proved to be a potential prognostic biomarker for some cancers in some studies [21,22]. Besides, miR-424 can suppress the metastasis and invasion of HCC [23]. To explore the role of miR-424-5p in HCC, qRT-PCR was used to detect the expression level of miR-424-5p in HCC cell lines. The results showed that miR-424-5p expression level was remarkably lower in HCC cell lines (HEP G2, Hep3B, SNU-182, SNU-387) than that in normal liver cell line THLE-3, and miR-424-5p was most significantly down-regulated in HEP G2 cells. Hence, HEP G2 cell line was chosen for further *in vitro* experiments (Fig 1C). Next, we investigated the role of miR-424-5p in the growth of HCC cells. qRT-PCR detected that the expression of miR-424-5p in HEP G2 cells transfected with miR-424-5p mimic was significantly up-regulated compared with control group, indicating a higher transfection efficiency (Fig 1D). CCK-8 proliferation assay demonstrated that the proliferative activity of HEP G2 cells was significantly decreased after overexpressing miR-424-5p (Fig 1E). Given the strict control of cell cycle over cell proliferation, flow cytometry was used to analyze cell cycle distribution, and it was found that HEP G2 cells transfected with miR-424-5p mimic arrested in G0/G1 phase (Fig 1F). Taken together, these findings confirmed that miR-424-5p expression was prominently down-regulated in HCC, which induced cell cycle arrest in G0/G1 phase to inhibit the proliferation of HCC cells.

### 2.2 E2F7 is highly expressed in HCC tissue and cells, and E2F7 overexpression promotes the growth of HCC cells

**Fig 2:**
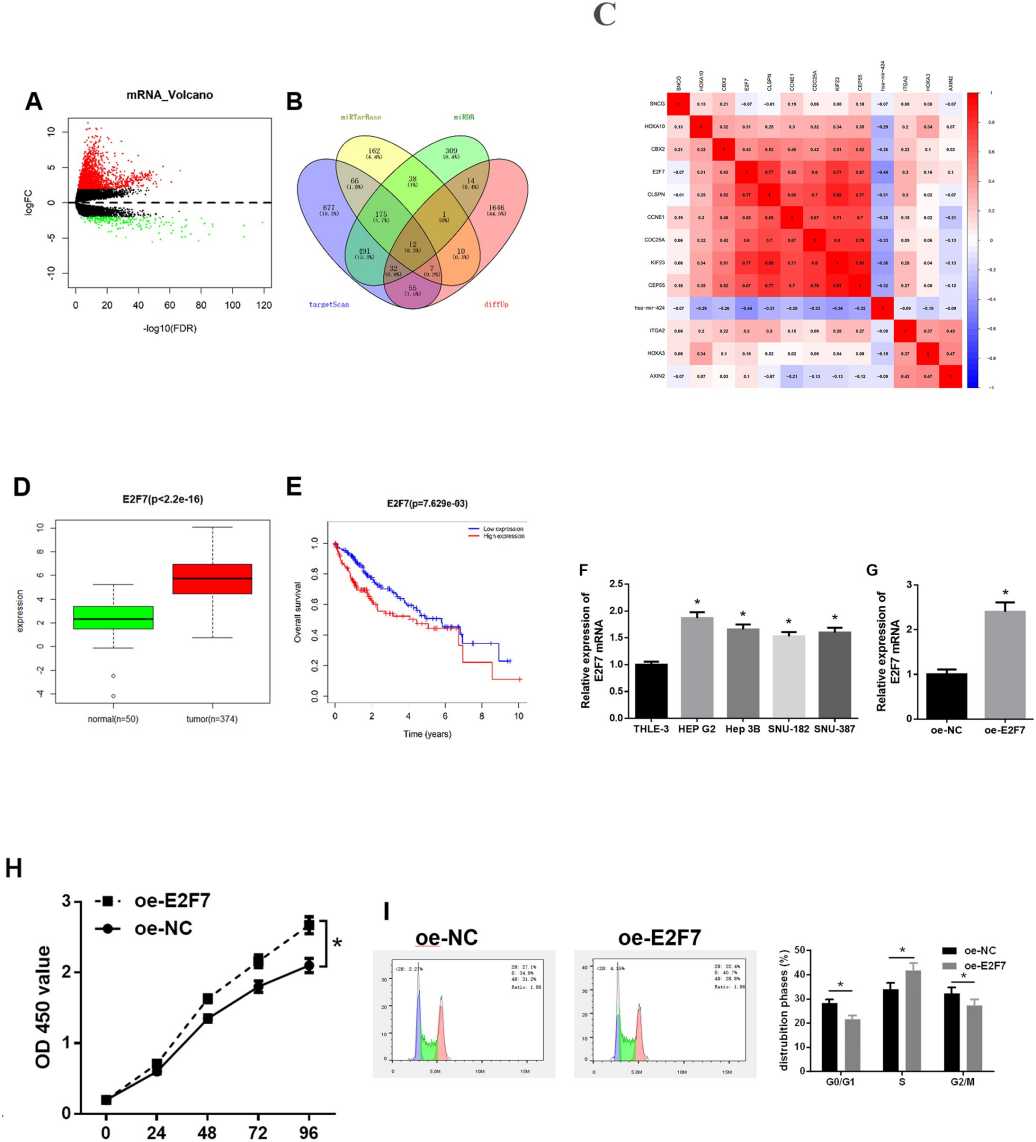
Expression level and function of E2F7 in HCC. A: Volcano plot of DEmRNAs in normal group and tumor group of HCC in the TCGA-LIHC dataset; B: Venn diagram of predicted target genes of miR-424-5p and upregulated DEmRNAs in TCGA; C: Pearson correlation analysis of miR-424-5p and its predicted candidate target genes; D: Box plot of the expression of E2F7 in normal group and tumor group; E: The survival curves were plotted to show the effect of E2F7 expression level on the prognosis of patients (red line represents high expression group, and blue line represents low expression group); F: The mRNA expression level of E2F7 in normal cell line THLE-3 and four HCC cell lines (HEP G2, Hep 3B, SNU-182 and SNU-387) was measured by qRT-PCR; G: The transfection efficiency of oe-E2F7 was measured by qRT-PCR; H: The proliferative activity of HEP G2 cells transfected with oe-E2F7 was detected by CCK-8 assay; I: The cell cycle distribution was analyzed by flow cytometry; * *p*<0.05.

A total of 1,984 DEmRNAs were screened using differential analysis on mRNA expression data from the TCGA-LIHC dataset (Fig 2A). Target gene prediction for miR-424-5p was conducted using TargetScan, miRDB and miRTarBase databases. Twelve DEmRNAs with binding sites of miR-424-5p were obtained from the intersection of 1,777 up-regulated DEmRNAs and predicted target genes (Fig 2B). Correlation analysis suggested a remarkable negative correlation between E2F7 and miR-424-5p (Fig 2C), and E2F7 was highly expressed in HCC tumor tissue (Fig 2D). Additionally, survival analysis of E2F7 revealed that highly expressed E2F7 was not conducive to the prognosis of patients (Fig 2E). It has been reported that E2F7 is implicated in cell cycle and proliferation of tumor cells under the regulation of miRNA [20,24]. In order to delve into the mechanism of E2F7 in HCC, we used qRT-PCR to determine its expression pattern in HCC cell lines and test the transfection efficiency of oe-E2F7. The results showed that the expression level of E2F7 in four HCC cell lines was extremely higher than that in normal liver line THLE-3. The up-regulated expression of E2F7 was the most evident in HEP G2 cells (Fig 2F). The expression level of E2F7 mRNA in HEP G2 cells of the oe-E2F7 group was 2.4 times higher than that of the control group (Fig 2G). CCK-8 assay suggested that overexpression of E2F7 significantly promoted the proliferative activity of HEP G2 cells (Fig 2H). The results of cell cycle assay indicated that overexpression of E2F7 promoted HEP G2 cell cycle from G0/G1 phase into S phase (Fig 2I). These findings illustrated that E2F7 was significantly up-regulated in HCC cells, and E2F7 induced cell cycle from G0/G1 into S phase and promoted cell proliferation of HCC cells.

### 2.3 E2F7 is a target of miR-424-5p

**Fig 3:**
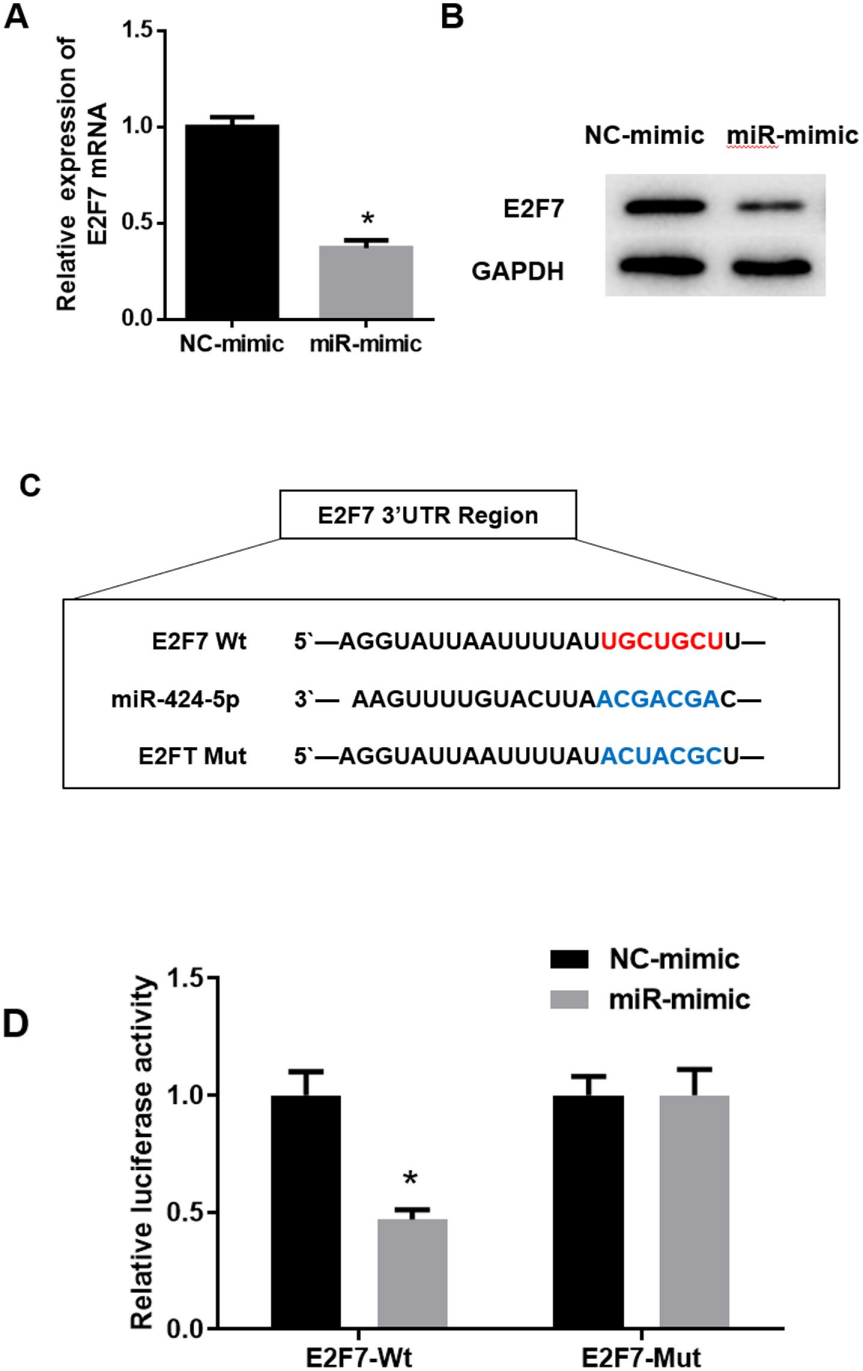
MiR-424-5p targets and inhibits the expression of E2F7. A: qRT-PCR was used to detect the effect of overexpression of miR-424-5p on the mRNA expression of E2F7; B: Western blot assay was performed to detect the effect of overexpressed miR-424-5p on E2F7 protein expression; C: The binding site sequences of E2F7-Wt/Mut and miR-424-5p; D: Dual-luciferase assay was used to detect the luciferase activity in E2F7-Wt and E2F7-Mut groups; * *p*<0.05.

Bioinformatics analysis suggested that E2F7 might be a target of miR-424-5p in HCC, so we conducted some experiments to verify it. qRT-PCR and western blot assays were utilized to examine whether the ectopic expression of miR-424-5p would affect the mRNA and protein expression levels of E2F7. The results showed that the mRNA and protein expression levels of E2F7 in the miR-mimic group were down-regulated in comparison to those in the NC-mimic group (Fig 3A and 3B), suggesting that overexpression of miR-424-5p could inhibit the expression of E2F7 in HCC cells. Subsequently, TargetScan was used to predict the binding sites of miR-424-5p on E2F7 (Fig 3C). Dual-luciferase assay was performed to validate whether there was a direct targeting relationship between them. It was exhibited that overexpression of miR-424-5p significantly decreased the relative luciferase activity of HEK 293t cells transfected with E2F7-Wt, while the luciferase activity of E2F7-Mut goup was unaffected (Fig 3D). In other words, miR-424-5p inhibited the expression of E2F7-Wt while had no effect on E2F7-Mut. These results fully elucidated that E2F7 was a direct target of miR-424-5p.

### 2.4 E2F7 overexpression reverses the inhibitory effect of miR-424-5p on cell proliferation of HCC via regulating cell cycle

**Fig 4:**
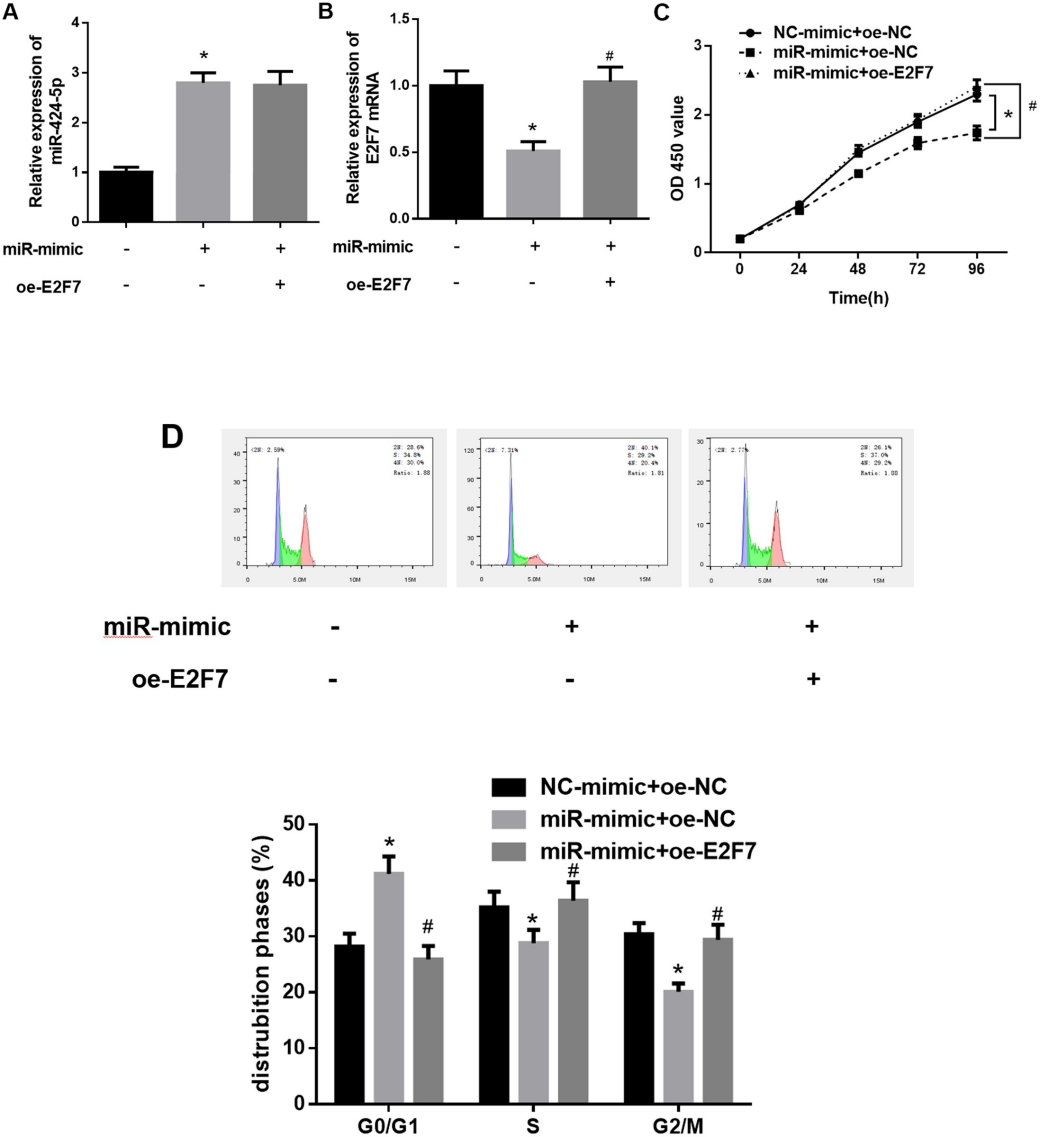
Overexpression of E2F7 reverses the inhibitory effect of miR-424-5p on HCC cells. A, B: qRT-PCR was used to detect miR-424-5p and E2F7 mRNA expression levels in different treatment groups; C: CCK-8 was used to detect the proliferative activity of HCC cells in different treatment groups; D: Flow cytometry was used to detect cell cycle distribution of HCC cells in different treatment groups; * means *p*<0.05 in comparison with NC-mimic+oe-NC groups, # means *p*<0.05 in comparison with miR-mimic + oe-NC group.

The regulatory effects of miR-424-5p and E2F7 on the proliferation of HCC cells and their targeting relationship had been confirmed in the above assays. It was reasonable to speculate that miR-424-5p could regulate cell cycle to affect cell proliferation of HCC cells by regulating E2F7 expression. Therefore, we divided HEP G2 cells into three groups, namely NC-mimic+oe-NC, miR-mimic+oe-NC and miR-mimic+oe-E2F7. The function of miR-424-5p/E2F7 in HCC cells was examined by rescue experiments. The results of qRT-PCR showed that miR-424-5p expression was markedly elevated while E2F7 was down-regulated in HEP G2 cells transfected with miR-mimic. The expression of E2F7 was significantly upregulated while that of miR-424-5p showed no obvious change in HEP G2 cells co-transfected with miR-mimic and oe-E2F7 in comparison with those in cells with miR-mimic only (Fig 4A and 4B). CCK-8 results indicated that compared to control group, the proliferative activity of HCC cells was clearly declined after overexpressing miR-424-5p, which was restored after overexpressing E2F7 (Fig 4C). The results of cell cycle assay revealed that miR-424-5p overexpression could block HEP G2 cells in G0/G1 phase, which was reversed when miR-424-5p and E2F7 were overexpressed simultaneously (Fig 4D). These findings validated that overexpression of E2F7 could counteract the phenotypes induced by miR-424-5p. In other words, miR-424-5p suppressed the growth of HCC cells by regulating E2F7.

## 3 Discussion

HCC is a type of primary malignant tumor and its high mortality rate poses a global challenge to improve clinical outcome under current medical circumstance [25,26]. It has been reported that the progression of HCC is determined by the interaction of dysregulated miRNAs and their target mRNAs [27]. MiR-424-5p was noted to be implicated in regulation of various physiological activities as an inhibitory factor of HCC in the previous research. Piao L *et al*. reported that miR-424-5p expression is reduced in HCC patients and induces apoptosis of HCC cells by targeting and inhibiting YAP1 expression [28]. Li D *et al*. observed that WEE1 is a target of miR-424-5p, and overexpression of WEE1 promotes the proliferation, migration and invasion of HCC cells [29]. Du H *et al*. believed that miR-424-5p acting as a tumor suppressor gene can inhibit the invasive ability of HCC cells by directly regulating TRIM29, showing its potential to be a novel prognostic indicator [30]. In accordance with the above studies, we found by bioinformatics analysis that miR-424-5p was a DEmiRNA which was stably expressed in HCC tissue. Then, it was detected that miR-424-5p was down-regulated in HCC cell lines. Results of CCK-8 and flow cytometry assays showed that miR-424-5p could inhibit cell proliferation and induce cells arrested in G0/G1 phase. Different from above studies, we found a new target E2F7 of miR-424-5p in HCC.

E2F family members are wildly expressed in various tissues and organs, and have been proved to regulate gene expression and participate in regulating cell proliferation, differentiation, DNA repair and cell cycle, etc. [31] E2F7 and E2F8 are the atypical members of the E2F family and are the only inhibitors in balance of the E2F transcription network, and E2F transcription network plays a critical role in embryonic development and control of E2F1-p53 apoptotic axis [32]. E2F8 is found to be significantly overexpressed in HCC, and it promotes the occurrence and development of HCC by activating E2F1/cyclin D1 signaling pathway to regulate the G1-S phase transition of cell cycle [33]. The overexpression of E2F7 in HCC tissue and cells was only reported in the study of Yu-Shui Ma *et al*. that can activate AKT 1-cyclin D1 signaling and the downstream cell cycle [20]. In this study, we used target gene prediction to speculate that E2F7 might be a potential target of miR-424-5p. Next, we used a series of *in vitro* experiments to detect E2F7 expression level and biological function. The results revealed that E2F7 was markedly up-regulated in HCC cell lines compared with that in normal liver cells, which could promote cell cycle from G1 to S phase in advance and further promote the proliferation of HCC cells. Then, we confirmed the targeted binding relationship between miR-424-5p and E2F7 through the dual-luciferase assay, and the rescue experiments showed that overexpression of E2F7 could reverse the inhibitory effect of miR-424-5p on the proliferation of HCC cells by regulating the cell cycle.

In brief, we found that miR-424-5p was down-regulated in HCC cells, and could inhibit the proliferation of HCC cells by blocking cells in G1 phase. The up-regulation of E2F7 could promote the proliferation of HCC cells. Moreover, dual-luciferase assay and rescue experiments were performed to explore the mechanism of the miR-424-5p/E2F7 regulatory axis in HCC. The results suggested that miR-424-5p suppressed the proliferation of HCC by targeting E2F7. The findings not only provide a complementary elaboration for the molecular mechanism underlying HCC occurrence and development, but also offer a reference for mining novel molecular pathways and target selection for the targeted therapy of HCC.

## Supporting information

S1 Fig (TIF)
