# Supplementary figures and images for: MiR-424-5p regulates cell cycle and inhibits proliferation of hepatocellular carcinoma cells by targeting E2F7
Source: PLoS One. 2020 Nov 17;15(11):e0242179. doi: 10.1371/journal.pone.0242179 (PMC7671513; doi:10.1371/journal.pone.0242179)

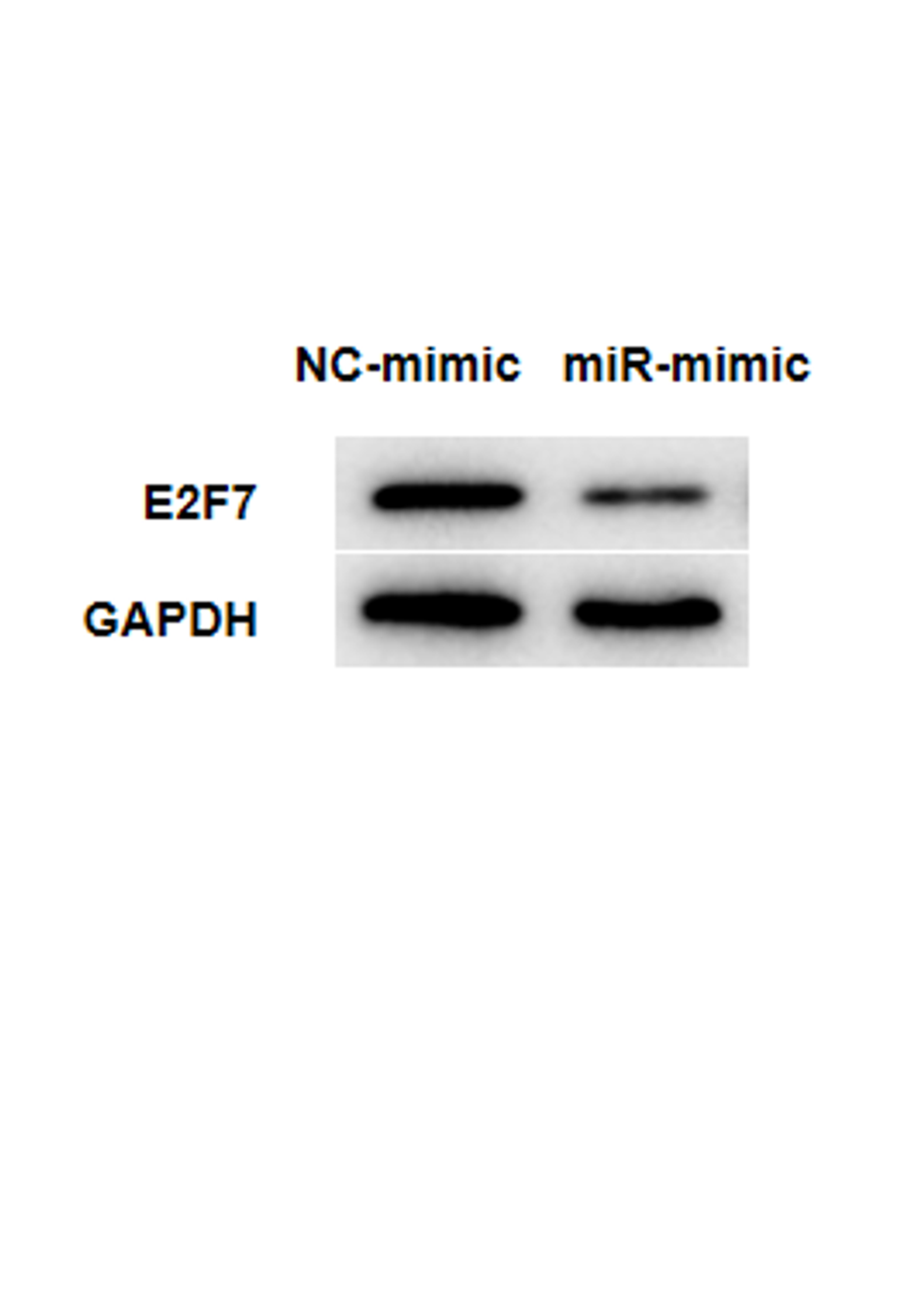

Supplement: S1 Fig — (TIF) [file pone.0242179.s001.tif]
